# Supplementary material for: An unexpected N-terminal loop in PD-1 dominates binding by nivolumab
Source: Nat Commun. 2017 Feb 6;8:14369. doi: 10.1038/ncomms14369 (PMC5303876; doi:10.1038/ncomms14369)
Supplement: Supplementary Information — Supplementary figures and supplementary tables. [file ncomms14369-s1.pdf]

Supplementary information

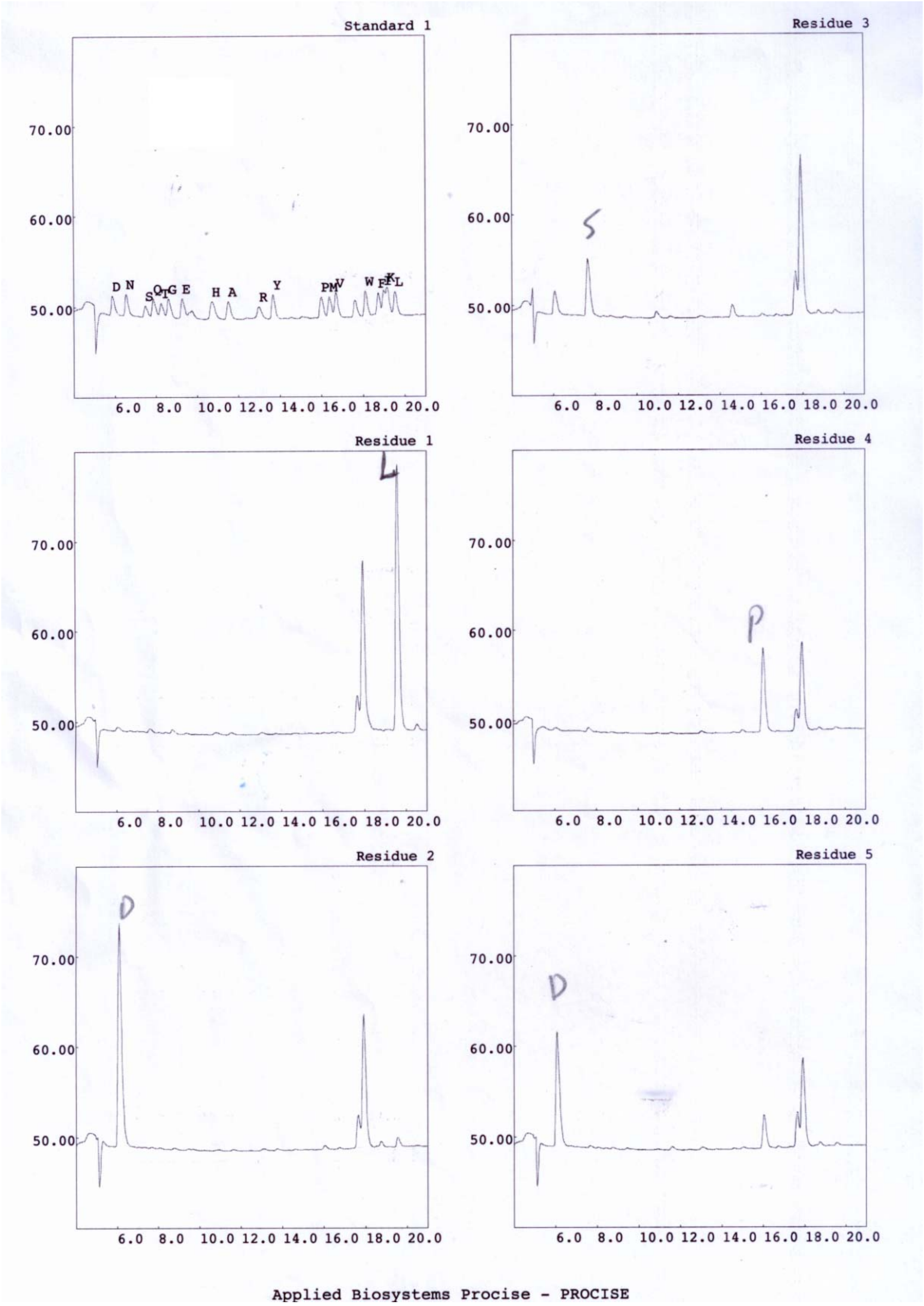

Supplementary Figure 1. N-terminal sequencing of PD-1 protein.

The first panel indicates the standard curve of each of the amino acid in Mass

Spectrometry. The following panels indicate the amino acid eluted following Edman digestion in each of the N-terninal residue. The characters labeled in each of the panel shows the most abundant amino acid at each of the residue.

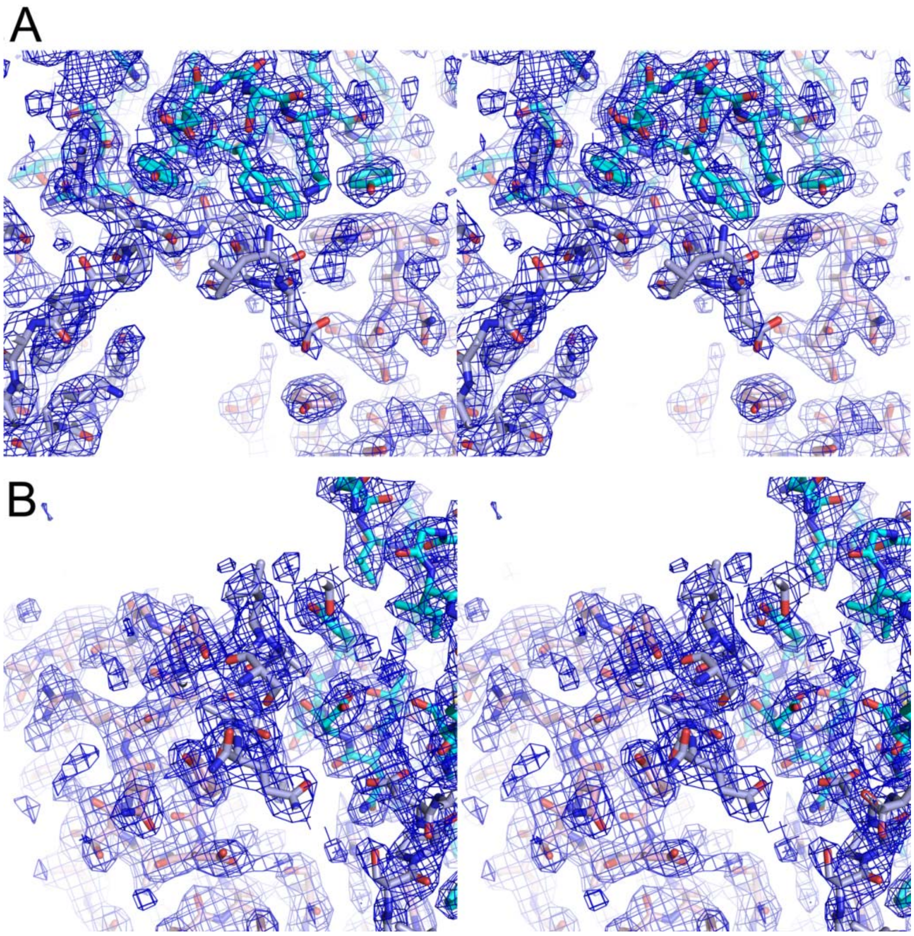

Supplementary Figure 2. A cross-eye stereo view (1.0 σ contour level) of the PD-1-nivolumab interface. Stereo view of the interface of nivolumab and N-loop (**A**) or FG-loop (**B**) of PD-1. PD-1 is colored in light blue while the heavy chain and light chain of nivolumab are colored in cyan and pink, respectively.

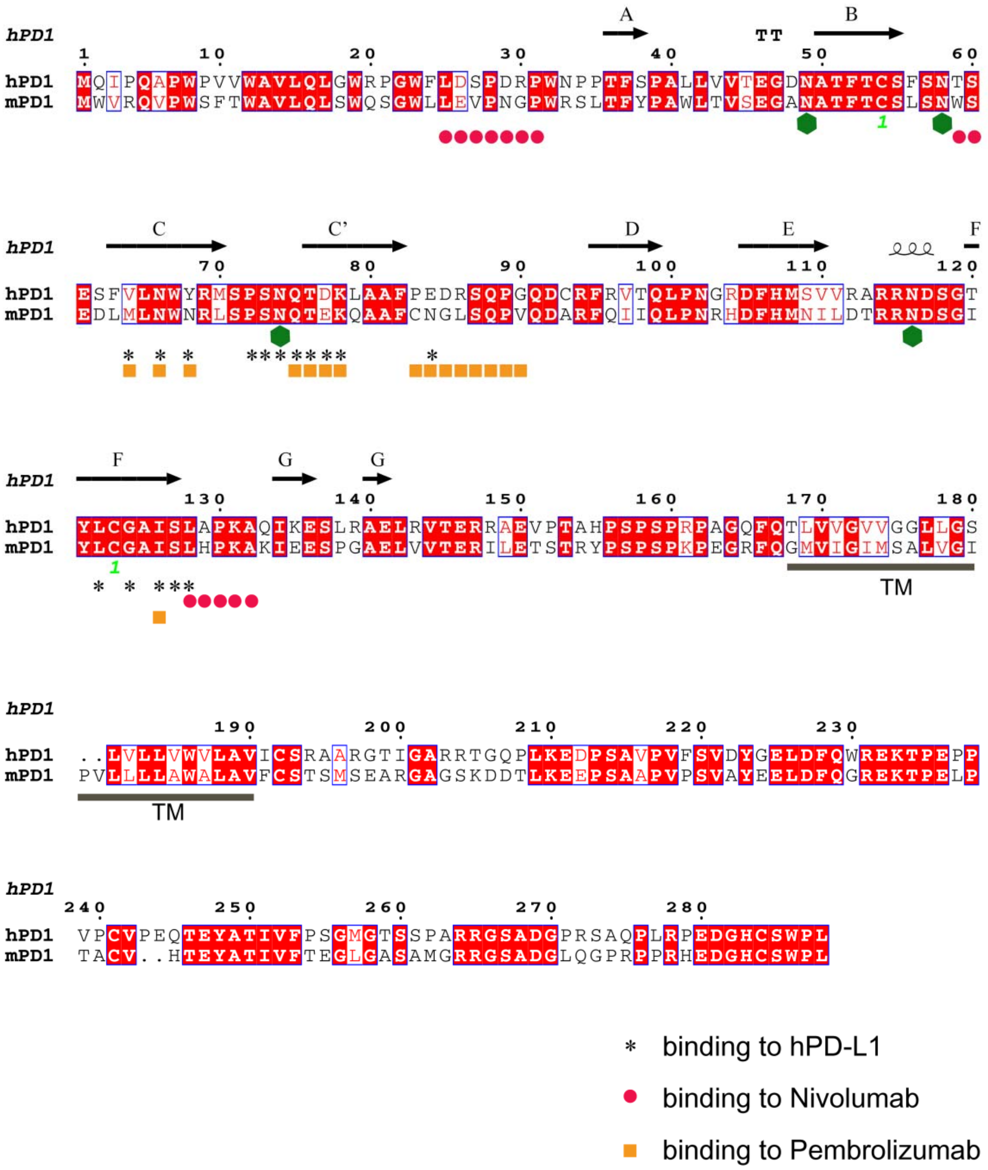

Supplementary Figure 3. Sequence alignment of human and murine PD-1. The sequence alignment indicates the amino acids contributing to PD-1-nivolumab or PD-1-pembrolizumab interaction. The solid green hexagon indicates the four potential glycosylation sites in the sequence. The number colored in green shows the amino acids that formed the intra-IgV disulfide bridge.

Supplementary Table 1. Interaction between nivolumab-Fab and PD-1

	Antibody	PD-1	Contacts	Total contacts[1]
H chain (V_H)	T28	T59,S60	5, 5	269
	S30	R30(2)[2]	8	
	N31	D29, R30(2), T59(1), S60	5, 19, 9, 1	
	S32	P28, D29	1, 6	
	G33	P28(1), D29(1)	5, 8	
	V50	P28	2	
	W52	L25(1), D26, S27(1), P28	8, 3, 10, 34	
	Y53	P28(1),D29,R30,P31	8,10,32,3	
	K57	L25(1)	6	
	N99	P28,D29,K131	7,12,4	
	D100	D29,A129,P130,K131(1)	1,6,6,12	
	D101	P130,K131(1)	9,3	
	Y102	L128,A129,P130	2,9,10	
L chain (V_L)	Y32	D26	2	68
	A34	K131	1	
	L46	P130, K131	3, 4	
	Y49	P130, K131, A132(1)	2, 15, 13	
	A55	P130	6	
	T56	L128,A129(1),P130(1),A132	4, 4, 11, 3	

[1] Numbers represent the number of atom-to-atom contacts between the antibody residues and the

hPD-1 residues, which were analyzed by the Contact program in CCP4 suite (the distance cutoff is 4.5Å).

[2] Numbers in the parentheses represent the number of hydrogen bonds between the antibody residues and the hPD-1 residues, which were analyzed by the Contact program in CCP4 suite (the distance cutoff is 3.5 Å).

Supplementary Table 2. Primers used for PD-1 site mutations

Primer	Sequence
N49A-F	TGACCGAAGGGGACGCTGCCACCTTCACCTG[1]
N49A-R	CAGGTGAAGGTGGCAGCGTCCCCTTCGGTCA
N74A-F	CGCATGAGCCCCAGCGCTCAGACGGACAAGCTG
N74A-R	CAGCTTGTCCGTCTGAGCGCTGGGGCTCATGCG
N116A-F	GTCAGGGCCCGGCGCGCTGACAGCGGCACCTAC
N116A-R	GTAGGTGCCGCTGTCAGCGCGCCGGGCCCTGAC
N58A-F	ACCTGCAGCTTCTCCGCTACATCGGAGAGCTTC
N58A-R	GAAGCTCTCCGATGTAGCGGAGAAGCTGCAGGT

[1] The red nucleotides indicate the site for alanine mutation

Supplementary Table 3. DNA sequence of nivolumab-Fab heavy chain and light chain

Gene name	Sequence
Nivolumab-Fab heavy chain	CAGGTCCAGCTGGTGGAGAGCGGTGGTGGTGTGGTGCAGCCCG GTCGTTCCCTGCGTCTGGATTGCAAAGCCTCCGGCATCACCTT CTCCAACAGCGGCATGCACTGGGTGCGTCAGGCTCCTGGTAAG GGCCTGGAGTGGGTGGCTGTGATCTGGTACGATGGCTCCAAGC GCTACTACGCCGACTCCGTGAAGGGTCGTTTCACCATCTCCCG CGACAACAGCAAGAACACCCTGTTCCTGCAGATGAACTCCCTG CGTGCCGAGGACACCGCCGTCTACTACTGCGCCACCAACGACG ACTACTGGGGTCAGGGCACTCTGGTGACCGTCTCCTCCGCTTC CACCAAGGGCCCCTCCGTGTTCCCTCTGGCTCCTTCCTCCAAG AGCACCAGCGGTGGCACTGCCGCCCTGGGTTGCCTGGTGAAGG ACTACTTCCCCGAGCCCGTGACCGTGTCCTGGAATAGCGGCGC TCTGACTTCCGGTGTGCACACTTTCCCCGCTGTCCTGCAATCC TCCGGTCTGTACTCCCTCTCCTCCGTGGTGACTGTGCCCAGCA GCTCCCTGGGCACCCAGACCTACATCTGCAACGTCAACCATAA GCCCTCCAACACCAAAGTCGACAAGAAAGTGGAGCCCAAGAGC

	TGCGACAAGCACCACCACCATCACCAC
Nivolumab-Fab light chain	GAATTCGAGATCGTGCTGACTCAGTCCCCTGCTACCCTGTCCC<br>TGTCCCCCGGTGAGCGTGCTACCCTGTCCTGCCGCGCTTCCCA<br>GAGCGTGTCCTCCTACCTGGCCTGGTATCAACAAAAGCCCGGC<br>CAAGCTCCCCGCCTGCTGATCTACGACGCCTCCAACCGCGCTA<br>CTGGCATCCCTGCTCGCTTCTCCGGTTCCGGTTCCGGCACTGA<br>CTTCACTCTGACCATCTCCTCCCTGGAGCCCGAGGATTTCGCT<br>GTGTACTACTGCCAGCAGTCCTCCAACTGGCCCCGTACCTTCG<br>GTCAGGGCACCAAGGTGGAGATCAAGCGCACCGTCGCTGCTCC<br>CTCCGTCTTCATCTTCCCTCCCTCCGACGAGCAGCTGAAGTCC<br>GGCACTGCCAGCGTCGTGTGCCTGCTGAACAACTTCTACCCCC<br>GCGAGGCTAAGGTGCAGTGGAAAGTGGACAACGCTCTGCAGTC<br>CGGCAACTCCCAAGAGTCCGTGACCGAGCAGGACTCCAAGGAC<br>AGCACCTACTCCCTGAGCTCCACCCTGACCCTCTCCAAGGCCG<br>ACTACGAGAAGCACAAGGTGTACGCCTGCGAGGTGACCCACCA<br>GGGTCTGTCCTCCCCCGTGACCAAGTCCTTCAACCGCGGTGAG<br>TGC
